# Realistic changes in monounsaturated fatty acids and soluble fibers are able to improve glucose metabolism

**DOI:** 10.1186/1758-5996-6-136

**Published:** 2014-12-07

**Authors:** Camila Risso de Barros, Adriana Cezaretto, Maíra Ladeia Rodrigues Curti, Milena Monfort Pires, Luciana Dias Folchetti, Antonela Siqueira-Catania, Sandra Roberta Gouvea Ferreira

**Affiliations:** Department of Epidemiology, School of Public Health, University of São Paulo, São Paulo, Brazil

**Keywords:** Monounsaturated fatty acids, Fiber intake, Lifestyle intervention, Glucose metabolism

## Abstract

**Background/objectives:**

Cardioprotective effects of Mediterranean-style diet have been shown. Instead of excluding foods, replacement or addition may facilitate compliance with impact on glucose metabolism of individuals at cardiometabolic risk. This study investigated the effect of changing selected nutrients intake on glucose metabolism during a lifestyle intervention tailored to living conditions of prediabetic Brazilians.

**Subjects/methods:**

183 prediabetic adults treated under the Brazilian public health system underwent an 18-month intervention on diet and physical activity. Dietary counseling focused on reducing saturated fat replaced by unsaturated fatty acids. Data were collected at baseline and after follow-up. ANOVA and multiple linear regression were used to test association of changes in nutrients intake with changes in plasma glucose.

**Results:**

Changes in fasting and 2-h plasma glucose but not in weight, HOMA-IR or C-reactive protein decreased after intervention across tertiles of MUFA changes (p-trend 0.017 and 0.024, respectively). Regression models showed that increase in MUFA intake was independently associated with reduction in fasting (β -1.475, p = 0.008) and 2-h plasma glucose (β -3.321, p = 0.007). Moreover, increase in soluble fibers intake was associated with decrease in fasting plasma glucose (β -1.579, p = 0.038). Adjustment for anthropometric measurements did not change the results but did after including change in insulin in the models.

**Conclusions:**

Increases of MUFA and soluble fibers intakes promote benefits on glucose metabolism, independently of adiposity, during a realistic lifestyle intervention in at-risk individuals. Mechanisms mediating these processes may include mainly insulin sensitivity improvement.

## Background

Obesity-related diseases, such as type 2 diabetes and cardiovascular disease, are major preventable problems which could be attenuated by a healthy diet and exercise [1, 2]. However, long-term compliance with a healthy lifestyle is a challenge, particularly in emerging countries, where populations have less access to healthy foods, usually more expensive, and fewer opportunities for exercising. Dietary recommendations have emphasized reduction in fat consumption and encouraged fiber intake [3]. The deleterious effects of saturated fatty acids (SFA) on cardiovascular risk profile obfuscated the benefits of unsaturated fat, whose cardioprotective role has been reinforced in more recent publications [4, 5]. Observational studies reported lower cardiovascular mortality rates in populations consuming high proportions of dietary unsaturated fatty acids and fibers [6]. Underlying mechanisms involve inflammatory mediators which interfere in glucose metabolism [7]. However, a diet rich in MUFA did not improve insulin sensitivity in healthy participants of a Nordic randomized clinical trial, except for a subgroup with a total fat intake < 37% of energy [8]. It remains unclear whether beneficial effects on glucose metabolism are independent of body weight [9, 10]. Also, for cardiovascular prevention, a mixture of soluble and insoluble fibers has been recommended. High-fiber diets have been associated with benefits on glucose and lipid metabolism and may lower risk of cardiovascular events [11]*.* Observational and prospective studies provided evidence of improvement in insulin sensitivity [12, 13] but the effect of fibers on inflammatory process is not yet well established [14].

Efficacy of the PREDIMED diet on reducing weight gain [15] and preventing cardiometabolic outcomes [16] has motivated several countries to change their eating habits. Low acceptance of this dietary pattern outside Mediterranean region was described in a Northern European population [17]. In Latinos living in South America, a better compliance with a diet rich in vegetable oil, whole grains, fruits and vegetables, could be expected considering their ancestry and local food availability.

Effectiveness of interventions on quality of fat and amount of dietary fiber, tailored to public health systems of developing countries, was scarcely investigated. In general, dietary interventions imply food restrictions; instead of excluding foods, replacement or even addition may be a better strategy to achieve metabolic benefits. We hypothesized that realistic changes in Brazilian eating habits may facilitate compliance with impact on glucose metabolism of individuals at cardiometabolic risk. This study investigated the effect of changing selected nutrients intake on glucose metabolism during a lifestyle intervention tailored to living conditions of prediabetic Brazilians.

## Subjects and methods

During 2008 and 2009, 438 individuals aged 18 to 79 years, treated under the public health system of the São Paulo city, Brazil, were screened for type 2 diabetes using a questionnaire adapted from the FINDRISC [18] and capillary glycemia. Individuals who seek this assistance tend to have low incomes and approximately 53% of the sample had up to 8 years of schooling. Selected individuals were invited to a clinical examination and laboratory tests including a 75-gram oral glucose tolerance test. Those with prediabetes (impaired fasting glycemia or impaired glucose tolerance) [19] were invited to participate in 18-month lifestyle interventions for diabetes prevention. Exclusion criteria were overt diabetes, medical history of neurological or psychiatric disturbances and thyroid, liver, renal and infectious diseases. The Institutional Ethics Committee approved the study and written consent was obtained from all participants. This trial was registered in the Brazilian registry center of the WHO International Clinical Trials Registry Platform (RBR #65 N292 at http://www.ensaiosclinicos.gov.br).

**Figure 1:**
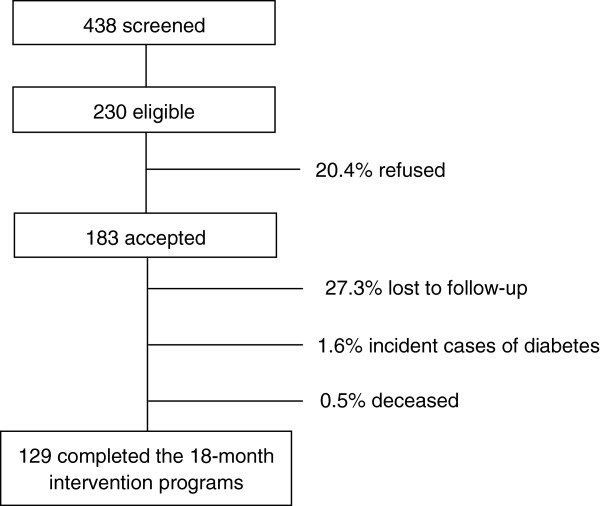
**Flowchart of individuals in each stage of the study.**

From 230 eligible individuals, 183 agreed to participate and 129 completed the intervention period until year 2010 (Figure 1). Among those who did not agree to participate there was a predominance of men; baseline socio-demographic, anthropometric and metabolic variables of non-participants did not differ from participants. Data of participants who were lost to follow-up were similar to those who completed the study. Drop-outs were mainly due to limited time to attend the visits during business hours.

### Study protocol

Participants were randomly assigned to one of two 18-month interventions on dietary habits and physical activity. Both interventions have quarterly medical consultations when counseling for changing lifestyle was reinforced but differed by the inclusion of psychoeducative sessions [20, 21]. Questionnaires and clinical data were collected at baseline and after follow-up. Baseline clinical data of the groups of participants were similar [22] and also the effects of the two types of interventions were comparable (data not shown). For the purpose of this study, the total sample was analyzed independent of the intervention type.

Dietary counseling was based on caloric restriction for those with elevated body mass index, reduction in foods rich in SFA (butter, whole milk and whole dairy products, visible fatty from meats and cold meats) encouraging those rich in unsaturated fatty acids (1 tablespoon of olive oil at lunch and another at dinner every day, at least 2 servings of fish per week, nuts and avocado whenever possible), as well as on high intake of soluble and insoluble fibers (at least 5 servings of fruits and vegetables per day, 1 or 2 servings of legumes and at least half of daily grains from whole-grain sources). Intervention goals were SFA intake ≤ 10% per day, total fiber intake ≥ 20 g/day, physical activity ≥150 minutes/week and weight loss ≥ 5% when indicated.

Three nonconsecutive 24-hour dietary recalls (two weekdays and one weekend day) were obtained by trained nutritionists, based in the USDA Multi-Pass Method [23]. The long version of the International Physical Activity Questionnaire was employed to assess physical activity level [24]. Blood pressure was measured at rest in sitting position by an automatic blood pressure device (Omron HEM-712C, Omron Health Care, USA). The average of the last two recorded measurements was used in analyses.

Participants were submitted to a 75-gram oral glucose tolerance test. Plasma glucose and lipoproteins were immediately determined and aliquots were frozen at -80°C for further determinations of inflammatory markers and hormones. Ultra sensitivity C-reactive (CRP) and tumor necrosis factor-alpha (TNF-α) concentrations were determined by immunoenzyme chemiluminescent assay (Immulite, Diagnostic Products Corporation, Los Angeles, CA, USA) and insulin by immunometric assay (AutoDelfia, Perkin Elmer Life Sciences Inc, Norton, OH, USA). Homeostasis model assessment (HOMA-IR) was used to assess insulin resistance [25].

### Statistical analysis

Household measurements of foods were transformed into measurement units and nutritional values calculated using the Nutrition Data System for Research (Nutrition Coordinating Center, Minneapolis, US). Participants with energy intake < 500 kcal were excluded from the analysis [26]. Mean nutrient intakes were expressed in grams and/or percent of total energy intake (% E). Dietary data were adjusted according to the method of Willett & Stampfer [27].

Data were expressed as means and standard deviations or percentages. Normality of variables was verified with histogram and Kolmogorov-Smirnov test. When distributions were skewed they were log-transformed before analysis and values were back-transformed to return to the natural scale. Student *t* test (or non-parametric equivalent) was used for comparisons of variables before and after intervention and Pearson’s coefficient to test correlations between changes in variables. Changes in nutrient intake were categorized into tertiles; ANOVA with Bonferroni’s method for pair comparisons was employed to compare changes in plasma glucose across tertiles and p-trend calculated. Dietary variables with a p-value <0.20 were selected for multivariate analysis. Stepwise linear regression was used to analyze association with changes in fasting or post-load plasma glucose (dependent variables), with adjustments for age, sex, change in leisure physical activity, change in SFA, family history of diabetes and type of intervention. Also, weight or waist circumference, CRP and insulin were included in final models to examine the influence of body adiposity, inflammation and insulin resistance mediating the relationship between dietary intake and glucose homeostasis. Coefficients and 95% confidence intervals were provided. Analyses were performed using Statistical Package for Social Sciences version 17.0 for Windows (SPSS Inc., Chicago, Illinois, USA). A p-value of <0.05 was considered significant.

## Results

Mean age of 183 participants (65% women) was 54.7 ± 12.3 years and 86% were overweight or obese at baseline. After intervention, reductions in total energy (1.822 ± 679 to 1.542 ± 548 kcal/day, p < 0.01), carbohydrates (51.0 ± 6.9 to 49.6 ± 6.7% of energy, p = 0.036), added sugars (36.8 ± 20.3 to 28.5 ± 20.8% of energy, p < 0.01) and *trans* fatty acids (3.3 ± 2.3 to 2.6 ± 2.2 g, p < 0.01) intakes were found, but not in unsaturated fat and fibers intakes and physical activity level (Table 1). Additionally, decreases in weight (78.7 ± 14.8 to 77.0 ± 14.9 kg, p < 0.01), waist circumference (101.1 ± 13.0 to 98.7 ± 12.3 cm, p < 0.01), mean blood pressure (109.7 ± 14.6 to 95.0 ± 10.1 mmHg, p < 0.01), fasting plasma glucose (98.7 ± 11.5 to 95.9 ± 12.1 mg/dL, p = 0.029), LDL-c (130.2 ± 40.5 to 121.3 ± 40 mg/dL, p = 0.023), HOMA-IR (2.49 ± 1.81 to 1.66 ± 0.91, p < 0.01) and inflammatory markers were detected as well as increase in HDL-c (42.5 ± 12.5 to 49.5 ± 14.6 mg/dL, p < 0.01).

**Figure 2:**
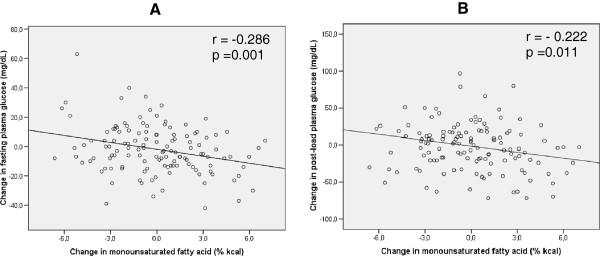
**Correlation of changes in MUFA with changes in fasting (panel A) and post-load plasma glucose (panel B).** MUFA – monounsaturated fatty acids. Pearson correlation test.

In average, MUFA intake was the same after intervention, since several participants increased but a proportion decreased consumption. Changes in MUFA intake were found to be inversely correlated to changes in fasting (r = -0.286; p = 0.001) and 2-hour plasma glucose (r = -0.222; p = 0.011) (Figure 2). Changes in other fatty acids and other nutrients intakes were not significantly correlated to changes in glucose levels.

**Table 1:** **Mean (± standard deviation) of dietary, physical activity and clinical data of participants at baseline and after intervention**

	Baseline	After intervention	P-value
• ***Daily habits variables***			
Energy (kJ and kcal/day)	7,626 ± 2,842 (1,822 ± 679)	6,455 ± 2,294 (1,542 ± 548)	<0.001
Carbohydrate (% E)	51.0 ± 6.9	49.6 ± 6.7	0.036
Added sugar (g)	36.8 ± 20.3	28.5 ± 20.8	<0.001
Protein (% E)	17.9 ± 3.5	19.9 ± 4.1	<0.001
Total fat (% E)	31.4 ± 5.5	31.3 ± 5.6	0.847
Saturated fatty acids (% E)	9.7 ± 2.9	9.6 ± 2.7	0.658
Monounsaturated fatty acids (% E)	10.8 ± 2.6	10.8 ± 2.7	0.951
Polyunsaturated fatty acids (% E)	8.2 ± 2.1	8.1 ± 2.0	0.724
*Trans* fatty acids (g)	3.3 ± 2.3	2.6 ± 2.2	<0.001
Soluble fibers/1,000 kcal (g)*	2.4 ± 1.3	2.2 ± 1.6	0.099
Unsoluble fibers/1,000 kcal (g)*	6.9 ± 2.8	6.9 ± 3.7	0.222
Leisure physical activity (min/week)*	36.6 ± 66.3	46.4 ± 82.0	0.466
• ***Clinical variables***			
Body weight (kg)	78.7 ± 14.8	77.0 ± 14.9	<0.001
Waist circumference (cm)	101.1 ± 13.0	98.7 ± 12.3	<0.001
Mean blood pressure (mmHg)	109.7 ± 14.6	95.0 ± 10.1	<0.001
Fasting plasma glucose (mg/dL)	98.7 ± 11.5	95.9 ± 12.1	0.029
2-h plasma glucose (mg/dL)	117.5 ± 27.7	113.2 ± 30.5	0.111
Triglycerides (mg/dL)	157.3 ± 67.2	149.3 ± 81.3	0.191
LDL-cholesterol (mg/dL)	130.2 ± 40.5	121.3 ± 40.0	0.023
HDL-cholesterol (mg/dL)	42.5 ± 12.5	49.5 ± 14.6	<0.001
HOMA-IR*	2.49 ± 1.81	1.66 ± 0.91	<0.001
C-reactive protein (mg/dL)*	0.34 ± 0.24	0.04 ± 0.05	<0.001
Tumor necrosis factor-α (pg/dL)*	12.5 ± 6.3	10.4 ± 6.8	<0.001

Student t test; *Wilcoxon test.

E, total energy intake.

Individuals who were diagnosed with diabetes during the intervention period were excluded from this analysis.

Significant trends to reductions in fasting and 2-hour plasma glucose after intervention across tertiles of MUFA changes (p-trend = 0.017 and 0.024, respectively) were verified but not in body weight, HOMA-IR or CRP concentration (Table 2). When stratifying according to tertiles of SFA, *trans* fatty acids or of fiber intake changes, no association was detected (data not shown).

**Table 2:** **Mean values of changes in clinical variables across tertiles of changes in MUFA intake (% energy)**

	Tertile 1	Tertile 2	Tertile 3	P-trend
	-6.6 to -1.3	-1.3 to 1.1	1.1 to 7.0	
Change in weight (kg)	-2.5 ± 4.0	-1.0 ± 3.8	-1.4 ± 3.8	0.151
Change in fasting plasma glucose (mg/dL)	3.1 ± 18.0	-1.6 ± 16.1	-6.9 ± 13.3^Ɨ^	0.017
Change in 2-h plasma glucose (mg/dL)	3.1 ± 40.1	5.9 ± 31.6	-13.5 ± 33.2^θ^	0.024
Change in HOMA-IR	-1.1 ± 1.9	-0.8 ± 1.5	-0.6 ± 1.5	0.448
Change in C-reactive protein (mg/dL)	-0.32 ± 0.23	-0.25 ± 0.19	-0.30 ± 0.24	0.273

ANOVA and Bonferroni’s method for pair comparisons.

^Ɨ^p < 0.05 *vs*. tertile 1 ^θ^p < 0.05 *vs*. tertile 2.

In multiple linear regression, increase in MUFA intake was associated with reduction in fasting [ß -1.90 (95% CI -3.08; -0.72)] and 2-h plasma glucose [ß -3.55 (95% CI -6.30; -0.80 (-4.78; -0.62)], adjusted for age, sex, change in leisure physical activity and in saturated fatty acids, family history of diabetes, type of intervention and changes in body weight, waist circumference and CRP levels (Table 3, Model 1 + Change in C-reactive protein). Moreover, increase in soluble fibers intake was independently associated with decrease in fasting plasma glucose only [ß -1.53 (-3.04; -0.02)]. All other changes in dietary variables were not associated with changes in plasma glucose. Additional adjustments for anthropometric variables changes did not modify the associations of MUFA and soluble fibers with plasma glucose. On the other hand, when changes in CRP were added, association of soluble fibers lost significance. Similarly, statistical significance disappeared adding changes in insulin to model 1 of MUFA and soluble fibers.

**Table 3:** **Association coefficients between intervention-induced changes in nutrients intake and in fasting and 2-h plasma glucose**

	Change in fasting plasma glucose		Change in 2-h plasma glucose	
	ß (95% CI)	p-value	ß (95% CI)	p-value
*Crude*				
Change in MUFA (% E)	-1.58 (-2.51; -0.65)	0.001	-2.70 (-4.78; -0.62)	0.011
Change in soluble fibers (g)	-0.45 (-1.71; 0.81)	0.481	-1.24 (-4.01; 1.54)	0.379
*Model 1*				
Change in MUFA (% E)	-1.48 (-2.55; -0.40)	0.008	-3.32 (-5.71; -0.93)	0.007
Change in soluble fibers (g)	-1.58 (-3.07; -0.09)	0.038	-1.88 (-5.19; 1.44)	0.264
*Model 1 + Change in weight*				
Change in MUFA (% E)	-1.48 (-2.57; -0.39)	0.008	-3.44 (-5.85; -1.04)	0.005
Change in soluble fibers (g)	-1.57 (-3.08; -0.06)	0.041	-1.71 (-5.05; 1.63)	0.311
*Model 1 + Change in waist circumference*				
Change in MUFA (% E)	-1.52 (-2.61; -0.42)	0.007	-3.68 (-6.07; -1.30)	0.003
Change in soluble fibers (g)	-1.53 (-3.04; -0.02)	0.047	-1.57 (-4.77; 1.23)	0.378
*Model 1 + Change in C-reactive protein*				
Change in MUFA (% E)	-1.90 (-3.08; -0.72)	0.002	-3.55 (-6.30; -0.80)	0.012
Change in soluble fibers (g)	-1.28 (-2.89; 0.33)	0.825	-2.54 (-6.28; 1.20)	0.181
*Model 1 + Change in 2-h insulin*				
Change in MUFA (% E)	-0.54 (-2.85; 1.76)	0.631	-3.20 (-8.27; 1.87)	0.205
Change in soluble fibers (g)	-0.84 (-3.45; 1.76)	0.719	0.20 (-5.13; 5.93)	0.943

Multiple linear regression analysis.

CI, confidence interval; MUFA, monounsaturated fatty acids; E, total energy intake.

Model 1: age, sex, change in leisure physical activity, change in saturated fatty acids, family history of diabetes (no or yes), type of intervention.

## Discussion

This real-life lifestyle intervention in at-risk individuals treated under the Brazilian public health system was able to improve dietary habits, inducing benefits in cardiometabolic profile. Our findings are relevant since they indicate that benefits previously described in efficacy trials for diabetes prevention from developed countries [2, 28] may be feasible in developing countries where projection of this disease is mainly alarming [29].

It was our interest to investigate which nutrients could most contribute to the improvement of glucose metabolism in order to orientate future public health policies. In agreement with prospective studies involving Mediterranean-style diets, our data on MUFA and soluble fiber intakes suggested that even small increments in these nutrients were able to induce favorable effects on glucose metabolism [5, 15, 16].

In contrast to the usual concern regarding SFA recommendation for individuals at high cardiovascular risk [2, 28], our most interesting finding was related to MUFA intake. Although increased mean MUFA intake could not be demonstrated in our sample, its protective effect on glucose metabolism was consistently suggested in all analyzes performed. The large intake range allowed examining how differences in MUFA intake were associated with changes in clinical outcomes. Even after adjustment for several variables, MUFA intake maintained associated with reductions in plasma glucose levels. Each increment of 5% energy of MUFA ingested results in -7.5 mg/dL and -16.5 mg/dL decreases in fasting and 2-h plasma glucose, respectively. We speculate that, in the long-term, such glycemic decreases – even within the non-diabetic range – could have a beneficial role on the natural history toward type 2 diabetes. Based on the observations that cardiovascular outcomes occurred in a continuum [30], the term dysglycemia – referring to near-normal fasting plasma glucose levels – has been used [31]. In this early stage, pathophysiological mechanisms, such as low-grade inflammation and insulin resistance, contribute to increase cardiometabolic risk.

Along the same line, clinical trials have previously showed that substitution of SFA by MUFA improves insulin sensitivity [8, 32]. Accordingly, the PREDIMED observed a 52% reduction in diabetes incidence among individuals submitted to Mediterranean diets [5]. In our multiple analysis, adjustment for change in insulin caused loss of significance on the effect of MUFA intake, suggesting that the beneficial effects on glucose metabolism may occur via improvement on insulin sensitivity.

In insulin-resistant individuals with several metabolic disturbances, a high-MUFA diet was considered a good option for nutritional management of glucose and lipid metabolism, as well as hepatic steatosis [33, 34]. Despite lack of MUFA increase, our intervention was able to reduce HOMA-IR, concomitantly with improvement in plasma glucose and lipids levels. This nutrient is shown to affect cell membrane fatty acids composition and function, including changes in membrane fluidity, ion permeability and insulin receptor affinity, which could enhance glucose uptake [35]. Animal studies described contrasting effects SFA and MUFA enriched diets on GLUT4 translocation and provided evidence on possible mechanisms explaining their impact on glucose metabolism [36, 37]. MUFA-induced improvement in insulin sensitivity seemed to be associated with a preserved IRS-1/PI3K insulin signaling. Considering that this pathway is known to be influenced by inflammatory mediators, we expected that this nutrient could attenuate circulating levels of CRP and/or TNF-α [38]. This hypothesis was not supported in our study since adjusting for CRP changes did not interfere on the MUFA impact in plasma glucose. How dietary fatty acids composition modifies insulin sensitivity in humans requires further investigations using more appropriate study design.

In multiple linear regression, the cardiometabolic protective role of dietary fiber was also explored. Such analysis indicated that each increment of 5 gram of soluble fibers ingested should result in a reduction of 8 mg/dL in fasting plasma glucose. Increase in fiber intake was a goal of our intervention [3, 39]. In agreement with our results, an analysis of a subset of participants of the PREDIMED study showed that reductions on fasting glucose were higher among individuals in the upper 20% of fiber intake [40]. Despite the health benefits of fiber have been consistently demonstrated, there are still controversies on which fiber type has the most protective role on diabetes prevention [41, 42].

Fiber-rich diets are usually less energy dense, richer in micronutrients, and bring a feeling of satiety sooner [39]. In relation to benefits of soluble fibers on the glucose metabolism, it has been proposed that their viscosity contributes to slow gastric emptying rates, digestion and the absorption of glucose, consequently reducing glycemic excursion and insulin secretion. The stability of plasma glucose and insulin levels may potentially contribute to enhancing peripheral insulin sensitivity [39, 43]. Moreover, effects induced by the presence of fiber in the stomach and duodenum interfere in glucagon-like peptide 1 secretion, which could affect not only insulin release, but also insulin sensitivity [44]. In the present study, the loss of significance of soluble fibers after adjustment for change in insulin concentration reinforces that this nutrient improve plasma glucose by interfering on insulin release and/or action. Furthermore, adjusting for change in CRP, the effect the soluble fibers was lost, suggesting that such benefit could be occurring via systemic inflammation. In fact, anti-inflammatory effect of fiber was previously reported [45], as well as the deterioration of insulin sensitivity induced by low-grade inflammation [46].

Noteworthy, the effects of soluble fibers and MUFA intake were not modified after addition of variables change in weight or in waist circumference in the models, supporting that the reduction in adiposity may not be the main factor responsible for the dietary-induced benefits on glucose metabolism found in the current study. In agreement with our findings, an intervention with Mediterranean diet (>40% of energy from fat and non-energy-restricted), without specific orientation to weight loss, reduced the frequencies of metabolic disturbances [47].

Although the reduction in SFA intake has been recommended to improve insulin sensitivity, association between change in SFA and change in plasma glucose was not found in the present study. Our results are in agreement with the LIPGENE which did not find beneficial effect on insulin sensitivity after reducing the amount of this type of fat [48].

On daily basis, soluble fibers and MUFA intake could be increased by the consumption of oat and olive oil. According to our findings, addition of two tablespoons of olive oil (26 g = 19 g of MUFA = 8.6% of total energy intake) in a 2000 kcal diet would reduce fasting plasma glucose in 12.7 mg/dL and 2-hour glucose in 28.6 mg/dL. Similarly, adding two tablespoons of oats (30 g = 1.74 g of soluble fibers) would decrease fasting plasma glucose in 2.7 mg/dL. Both changes would be able, therefore, to reduce fasting glucose in 15.4 mg/dL and 2 h-plasma glucose in 28.6 mg/dL. We believe that these changes in the eating habits are feasible, even in middle-income countries. Adding a new food in dietary plan may be easier than restricting (such as restriction of SFA sources). Also, we speculate that soluble fibers and MUFA-rich foods in the meals could enhance satiety minimizing energy intake and facilitating body weight control.

Other mechanisms have been more recently proposed to explain relationships between diet changing and glucose metabolism, which might be mediated by the gut microbiota [49]. Understanding how dietary changes could shape this microbial ecosystem and then interact with host’s metabolism altering insulin sensitivity may contribute to orient lifestyle interventions.

Our study has limitations; some are related to intrinsic weakness of the available techniques to quantify food intake. However, several precautions were taken to improve the accuracy of the dietary data collected. We cannot exclude that different populations respond distinctly to the same nutrient modification in part due to genetic characteristics. Our study did not include a large sample, but has the strength of having a longitudinal design, conducted in an at-risk miscigenated population of a developing country. Despite the MUFA intake have not been an intervention goal, our results call attention to the importance of this fatty acid in the context of fat intake recommendations to individuals at cardiometabolic risk. Further lifestyle interventions designed for high-risk populations should consider increasing MUFA intake as dietary goal.

In summary, we conclude that increases of MUFA and soluble fibers intakes promote benefits on glucose metabolism, independently of adiposity, during a realistic lifestyle intervention in at-risk individuals. Possible mechanisms mediating these processes may mainly include improvement in insulin sensitivity. Encouraging fiber and MUFA intakes may be a good strategy for at-risk individuals without necessarily restricting energy consumption.
